# Pleiotropic Associations of Risk Variants Identified for Other Cancers With Lung Cancer Risk: The PAGE and TRICL Consortia

**DOI:** 10.1093/jnci/dju061

**Published:** 2014-03-28

**Authors:** S. Lani Park, Megan D. Fesinmeyer, Maria Timofeeva, Christian P. Caberto, Jonathan M. Kocarnik, Younghun Han, Shelly-Ann Love, Alicia Young, Logan Dumitrescu, Yi Lin, Robert Goodloe, Lynne R. Wilkens, Lucia Hindorff, Jay H. Fowke, Cara Carty, Steven Buyske, Frederick R. Schumacher, Anne Butler, Holli Dilks, Ewa Deelman, Michele L. Cote, Wei Chen, Mala Pande, David C. Christiani, John K. Field, Heike Bickebӧller, Angela Risch, Joachim Heinrich, Paul Brennan, Yufei Wang, Timothy Eisen, Richard S. Houlston, Michael Thun, Demetrius Albanes, Neil Caporaso, Ulrike Peters, Kari E. North, Gerardo Heiss, Dana C. Crawford, William S. Bush, Christopher A. Haiman, Maria Teresa Landi, Rayjean J. Hung, Charles Kooperberg, Christopher I. Amos, Loïc Le Marchand, Iona Cheng

**Affiliations:** **Affiliations of authors:**Department of Preventive Medicine, Norris Comprehensive Cancer Center, Keck School of Medicine, University of Southern California, Los Angeles, CA (SLP, FRS, CAH); Public Health Sciences Division, Fred Hutchinson Cancer Research Center, Seattle, WA (MDF, JMK, AY, YL, UP, CC, CK); Colon Cancer Genetics Group, Institute of Genetics and Molecular Medicine, University of Edinburgh and Medical Research Council Human Genetics Unit, Edinburgh, UK (MT); Epidemiology Program, University of Hawaii Cancer Center, Honolulu, HI (CPC, LRW, LL); Community and Family Medicine, Geisel School of Medicine, Dartmouth College, Hanover, NH (YH, CIA); Department of Epidemiology (S-AL, AB, KEN, GH) and Carolina Center for Genome Sciences (KEN), University of North Carolina, Chapel Hill, NC; Molecular Physiology and Biophysics (LD, DCC), Center for Human Genetics Research (LD, RG, HD, DCC, WSB), Vanderbilt Epidemiology Center (JHF), and Biomedical Informatics (WSB), Vanderbilt University, Nashville, TN; Division of Genomic Medicine, National Human Genome Research Institute (LH), and Division of Cancer Epidemiology and Genetics, National Cancer Institute (DA, NC, MTL), National Institutes of Health, Bethesda, MD; Department of Statistics and Biostatistics, Rutgers University, Piscataway, NJ (SB); Information Sciences Institute, University of Southern California, Marina del Rey, CA (ED); Department of Oncology, School of Medicine, Wayne State University, Detroit, Michigan (MLC); M.D. Anderson Cancer Center, Houston, TX (WC, MP); Harvard University School of Public Health, Boston, MA (DCC); Roy Castle Lung Cancer Research Programme, Department of Molecular and Clinical Cancer Medicine, The University of Liverpool Cancer Research Centre, Institute of Translational Medicine, The University of Liverpool, Liverpool, UK (JKF); Department of Genetic Epidemiology, University Medical Centre Göttingen, Göttingen, Germany (HB); DKFZ–German Cancer Research Center and Translational Lung Research Centre Heidelberg, Member of the German Center for Lung Research, Heidelberg, Germany (AR); Institute of Epidemiology I, Helmholtz Zentrum Munchen, Munich, Germany (JH); International Agency for Research on Cancer, Lyon, FR (PB); Division of Genetics and Epidemiology, Institute of Cancer Research, Sutton, Surrey, UK (YW, RSH); Cambridge University Health Partners, Cambridge, UK (TE); American Cancer Society, Epidemiology and Surveillance Research, Atlanta, GA (MT); Samuel Lunenfeld Research Institute of Mount Sinai Hospital, Toronto, ON (RJH); Cancer Prevention Institute of California, Fremont, CA (IC).; * For the Transdisciplinary Research in Cancer of the Lung Research Team.

## Abstract

**Background:**

Genome-wide association studies have identified hundreds of genetic variants associated with specific cancers. A few of these risk regions have been associated with more than one cancer site; however, a systematic evaluation of the associations between risk variants for other cancers and lung cancer risk has yet to be performed.

**Methods:**

We included 18023 patients with lung cancer and 60543 control subjects from two consortia, Population Architecture using Genomics and Epidemiology (PAGE) and Transdisciplinary Research in Cancer of the Lung (TRICL). We examined 165 single-nucleotide polymorphisms (SNPs) that were previously associated with at least one of 16 non–lung cancer sites. Study-specific logistic regression results underwent meta-analysis, and associations were also examined by race/ethnicity, histological cell type, sex, and smoking status. A Bonferroni-corrected *P* value of 2.5×10^–5^ was used to assign statistical significance.

**Results:**

The breast cancer SNP *LSP1* rs3817198 was associated with an increased risk of lung cancer (odds ratio [OR] = 1.10; 95% confidence interval [CI] = 1.05 to 1.14; *P* = 2.8×10^–6^). This association was strongest for women with adenocarcinoma (*P* = 1.2×10^–4^) and not statistically significant in men (*P* = .14) with this cell type (*P*_*het by sex*_ = .10). Two glioma risk variants, *TERT* rs2853676 and *CDKN2BAS1* rs4977756, which are located in regions previously associated with lung cancer, were associated with increased risk of adenocarcinoma (OR = 1.16; 95% CI = 1.10 to 1.22; *P* = 1.1×10^–8^) and squamous cell carcinoma (OR = 1.13; CI = 1.07 to 1.19; *P* = 2.5×10^–5^), respectively.

**Conclusions:**

Our findings demonstrate a novel pleiotropic association between the breast cancer *LSP1* risk region marked by variant rs3817198 and lung cancer risk.

Globally, lung cancer is the most common malignancy and cause of cancer-related deaths (1,2). Tobacco smoking is the primary risk factor for lung cancer, but there is evidence that genetic susceptibility plays a role. Notably, recent genome-wide association studies (GWASs) of lung cancer have identified single-nucleotide polymorphisms (SNPs) in at least 10 independent loci (*P* < 5×10^–8^) influencing risk in different populations (3). However, these identified loci explain only a small fraction of lung cancer susceptibility and the challenge remains to identify the many additional common risk loci that are expected to have small genetic effects (3).

To date, more than 400 SNPs have been associated with cancer in GWASs (3). The discovery of pleiotropic effects, where a single gene variant is associated with more than one phenotype, may allow for the identification of shared disease pathways. For cancer, this may ultimately lead to the detection of susceptible individuals as well as in the development of regimens for the prevention of multiple cancers and pathway-based treatment. Genetic variants at chromosome 8q24, in *TP53*, and in *TERT*, the telomerase reverse transcriptase gene, are examples of loci with pleiotropic effects for multiple cancer sites and other chronic diseases (4–6). For lung cancer, a systematic evaluation of possible pleiotropic associations for the many risk variants identified with other cancer sites has yet to be conducted.

These genetic associations may have been missed in prior GWASs of lung cancer due to the heavy multiple comparison penalty in surveying the entire genome or due to disease heterogeneity in factors such as histological cell types or smoking status. For example, *TERT* rs2736100 (7–9) has been primarily associated with risk of adenocarcinoma of the lung, often diagnosed among nonsmokers, whereas SNPs in the 15q25 region, which include nicotinic acetylcholine receptor genes involved in nicotine dependence, have been primarily associated with lung cancer among smokers (10).

Here, we examined the pleiotropic effects of 165 risk variants initially identified for other cancers on lung cancer risk. Our study included a collaboration between two large consortia (11,12), in which we also examined the consistency of associations by race/ethnicity, tumor histology, sex, and smoking status.

## Methods

### Study Participants

Two consortia contributed data to this study: the Population Architecture using Genomics and Epidemiology (PAGE) (12) and the Transdisciplinary Research in Cancer of the Lung (TRICL) (11), which is part of the Genetic Associations and MEchanisms in ONcology (GAME-ON) consortium, and is associated with the International Lung Cancer Consortium (ILCCO). This collaboration provided information on 18023 patients with lung cancer and 60543 control subjects from 13 studies (Supplementary Table 1, available online). Details regarding these participating studies are described in the Supplementary Data (available online). All studies were based on primary incident nonsarcoma and nonlymphoma lung cancer cases, and more than 95% of the cases were pathologically confirmed. The majority of these studies utilized patients and control subjects who had no history of another cancer. Among the few studies in which a small proportion of patients and control subjects had a history of another cancer, our findings were similar when excluding these participants. Participants’ informed consent and institutional review board approval was obtained for all studies except Epidemiologic Architecture for Genes Linked to Environment, which accesses the Vanderbilt University biorepository (EAGLE-BioVU), which is considered nonhuman subjects research due to sample de-identification (13).

### SNP Selection and Genotyping

A total of 165 SNPs associated with 16 malignancies excluding lung cancer and smoking-related SNPs were selected as of January 2010 from the National Human Genome Research Institute GWAS catalog (3) and review of the cancer GWASs and fine-mapping literature review (Supplementary Table 3, available online). Additionally, we studied 18 lung cancer risk variants to replicate their associations with lung cancer risk in populations of European ancestry (Supplementary Table 2, available online) (11). The risk allele for each SNP was defined as the allele associated with an increased risk of cancer in the initial report. For PAGE, candidate SNP genotyping was performed using Illumina BeadXpress (Women’s Health Initiative [WHI]), Sequenom (EAGLE-BioVU), and the TaqMan OpenArray platform (Multiethnic Cohort study [MEC]). Atherosclerosis Risk in Communities Study [ARIC] (in PAGE) and TRICL extracted genotypes from GWAS data and were comprised of only European-ancestry populations. The ARIC samples were genotyped using the Affymetrix 6.0 platform. Genotypes were called with Birdseed and only SNPs with call rate equal to or greater than 90%, MAF equal to or greater than 1%, and Hardy-Weinberg equilibrium *P* > 1×10^–6^ were considered for imputation. Untyped and missing SNPs were imputed using Mach1 v1.00.16 based on HapMap release 2 (build 36) and a European ancestry (CEU) reference panel (14). Imputed SNPs with a quality threshold of *r* ^2^ greater than or equal to 0.3 were included in this analysis. MEC, EAGLE-BioVU, and WHI could not impute missing SNPs due to the reduced number of variants genotyped. For TRICL, genotyping was performed using the Illumina HumanHap300 BeadChips or Human Hap550 or 610 Quad arrays. At the time of this analysis, imputed SNPs were not available for TRICL.

All PAGE studies, with the exception of ARIC, genotyped a panel of 128 ancestry informative markers (15) and used principal components analysis to estimate principal components of genetic ancestry (16). ARIC (17) and TRICL (11) estimated principal components of genetic ancestry based on GWAS data using EIGENSTRAT (16). These principal components of genetic ancestry were included in regression models to adjust for population substructure.

Standard quality-assurance and quality-control measures were utilized to ensure genotyping quality. In PAGE (12), samples and SNPs were included based on call rates (≥90%), concordance of blinded replicates (>98%), and departures from Hardy-Weinberg equilibrium (*P* < .001). More than 97.9% of samples and more than 99% of SNPs had a call rate equal to or greater than 95% in all four PAGE studies. In TRICL (11), samples were excluded if the average call rate was less than 90%; if there was sex discrepancy (threshold of heterozygosity >10% for men and <20% for women), unexpected duplicates, evidence of first-degree relatedness, or heterozygosity rates for autosomal chromosomes exceeding six standard deviations of the mean; samples with less than 80% European ancestry based on STRUCTURE (18) analysis, and outliers based on principal component analysis using EIGENSTRAT (16), were also excluded.

### Statistical Analyses

For each study, we estimated the association between each SNP and risk of lung cancer using unconditional logistic regression and an additive genetic model of the risk allele. Models were adjusted for age, sex, country/study center (as appropriate), principal components of genetic ancestry, and smoking status (never, former, current). The Liverpool and Institute of Cancer Research (ICR) studies, which used generic control subjects, were not adjusted for age, sex, or smoking status. Studies with more than 85 lung cancer cases per racial/ethnic group were retained for race/ethnicity-stratified analysis. Associations by tumor histology were estimated based on logistic models of World Health Organization–defined histological cell type (adenocarcinoma, squamous cell carcinoma [SCC], and small cell lung cancer) compared to all control subjects. Large cell lung cancers were not included in the histology-specific analysis due to their limited sample size and heterogeneous nature. Stratified analyses by sex and smoking status (never and ever) were also performed.

To examine whether the associations with SNPs in *TERT* were independent of the known lung cancer risk variant in *TERT* (rs2736100) (7), conditional analysis was performed.

The regression estimates were combined across studies using inverse-variance weighted, fixed-effect meta-analysis using the METAL program, tool for meta-analysis genomewide association scans (19). The Cochran Q statistic was used to test for heterogeneity by study and whether the meta-analyzed odds ratios (ORs) were heterogeneous by race/ethnicity, histological cell type, sex, and smoking status. To account for multiple testing of 165 SNPs and 11 stratified analyses (four race/ethnicities, three histological cell types, two sexes, two levels of smoking status), we used a Bonferroni-corrected *P* value to assign statistical significance (α = .05/[165 SNPs*12 above mentioned tests] = 2.5×10^–5^). No additional associations were detected at a less stringent *P* value (e.g., .05/165 SNPs = 3×10^–4^). Statistical tests were two-sided.

## Results

The main characteristics of the 18023 patients with lung cancer and 60543 control subjects are presented in Supplementary Table 1 (available online). The PAGE study was comprised of European-ancestry, African American, Hispanic, Asian, Pacific Islander, and American Indian populations. The TRICL study was comprised only of individuals of European descent. The great majority (96%) of subjects were of European ancestry. Also, the majority of patients and control subjects were older than 50 years, with the exception of the Helmholtz-Gemeinschaft Deutscher Forschungszentren Lung Cancer GWAS (HGF) Germany study, where all subjects were 50 years of age or younger (3%). All studies, except WHI, were comprised of both sexes. In all studies, patients were more likely to be ever smokers and control subjects were more likely to be never smokers. Histology information was available for all studies, with the exception of ARIC. Among the studies with histology information, adenocarcinoma (34.0%) was the most common cell type, with the exception of the International Agency for Research on Cancer (IARC) GWAS, where SCC was more common (35.6%).

We evaluated the association between 18 known lung risk variants located in previously identified lung cancer risk loci and risk of lung cancer among European-ancestry populations (Supplementary Table 2, available online). Of the 18 lung cancer risk variants, 16 replicated at *P* < .05.

**Figure 1.**
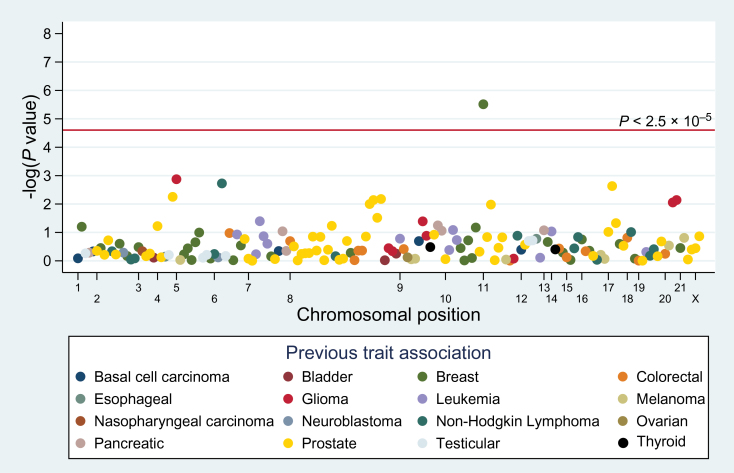
Manhattan plot of the meta-analysis association between risk variants of 16 other cancers and lung cancer. The solid line is the Bonferroni-corrected significance threshold. Each association is colored according to the cancer for which the single-nucleotide polymorphism was originally reported, and positioned on the *x*-axis according to its genomic position.

Among the 165 risk variants, 15 were nominally associated with lung cancer at *P* < .05 (Figure 1; Supplementary Table 3, available online), which is notably more than the eight associations expected by chance (i.e., 165 SNPs*.05 = 8.3). Using a binomial distribution with a *P* = .05 and n = 165 SNPs, the probability of observing 15 or more associations is .009. These 15 associations included eight prostate cancer variants, four glioma variants, one breast cancer variant, one childhood acute lymphocytic leukemia variant, and one follicular lymphoma variant. Twelve of the 15 SNPs were associated with an increased risk of lung cancer in the same direction of the known GWAS association. No heterogeneity by race/ethnicity (*P* > .05) was noted for the 15 nominally associated SNPs (Supplementary Table 4, available online).

**Figure 2.**
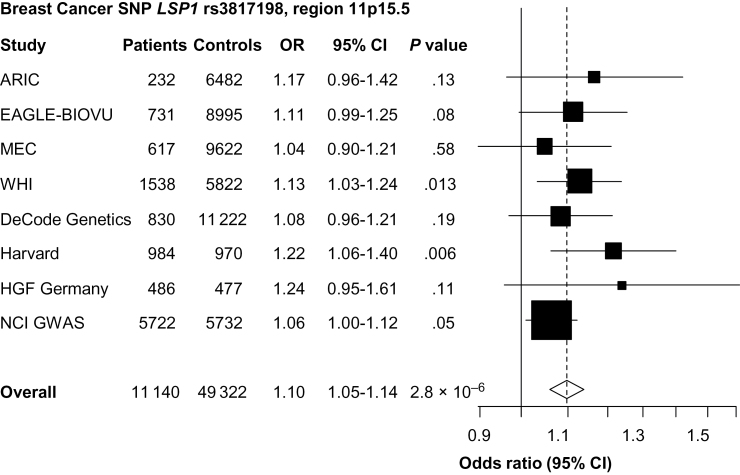
Forest plot of the association between lymphocyte-specific protein 1 (*LSP1*) rs3817198 and lung cancer risk. Study-specific and meta-analysis associations are plotted, modeling the C risk allele for breast cancer. Squares represent odds ratios (ORs); size of the square represents inverse of the variance of the log ORs; horizontal lines represent 95% confidence intervals (CIs); diamonds represent summary estimate combining the study-specific estimates with a fixed-effects model; solid vertical lines represent OR = 1; dashed vertical lines represent the overall ORs. The single-nucleotide polymorphism (SNP) rs3817198 was genotyped in all studies. GWAS = genome-wide association study.

The breast cancer SNP *LSP1* rs3817198 was associated with an increased risk of lung cancer (OR = 1.10; 95% confidence interval [CI] = 1.05 to 1.14) and remained statistically significant (*P* = 2.8×10^–6^) after correction for multiple comparisons (Figure 2). This association was heterogeneous by cell type (*P*_het_ = .03) and sex (*P*_het_ = .01), where it appeared to be limited to adenocarcinoma (OR = 1.11; 95% CI = 1.05 to 1.17; *P* = 1.14×10^–4^) (Supplementary Table 5, available online) and women (OR = 1.16; 95% CI = 1.09 to 1.23; *P* = 4.31×10^–6^) (Supplementary Table 6, available online). This association was not observed in SCC or small cell carcinoma (*P* ≥ .35) or in men (*P* = .16). In stratified analysis by both sex and histology cell type (data not shown), among studies with available data, we found that the association was present for female adenocarcinoma (n = 1,607 cases, 4 studies: EAGLE-BioVU, MEC, National Cancer Institute Lung GWAS (NCI), and WHI; OR = 1.19; *P* = 1.2×10^–4^). This association was not observed for male adenocarcinoma (n = 1507, 3 studies: EAGLE-BioVU, MEC, NCI; *P* = .14). However, the test for heterogeneity in effects between rs3817198 and adenocarcinoma by sex was not statistically significant (*P* = .10).

**Figure 3.**
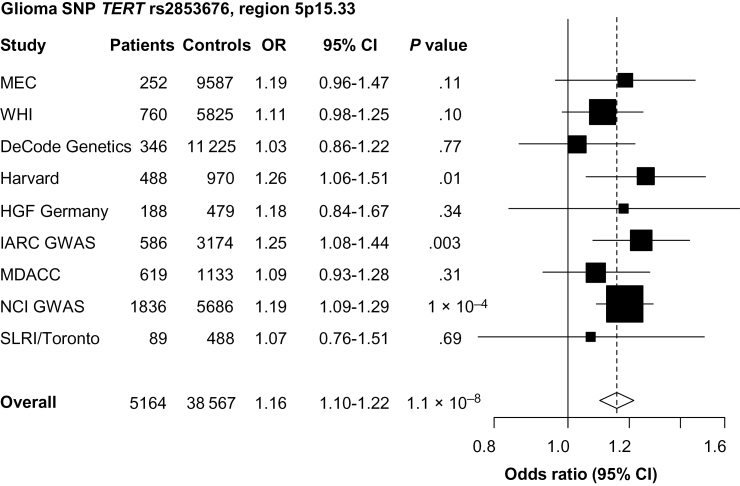
Forest plot of the association between telomerase reverse transcriptase gene (*TERT*) rs2853676 and lung adenocarcinoma risk. Study specific and meta-analysis associations are plotted, modeling the A risk allele for glioma. Squares represent odds ratios (ORs); size of the square represents inverse of the variance of the log ORs; horizontal lines represent 95% confidence intervals (CIs); diamonds represent summary estimate combining the study-specific estimates with a fixed-effects model; solid vertical lines represent OR = 1; dashed vertical lines represent the overall ORs. The single-nucleotide polymorphism (SNP) rs2853676 was genotyped in all studies. GWAS = genome-wide association study.

Whereas the *TERT* rs2853676 variant was only nominally associated with overall lung cancer (*P* = .001) (Supplementary Table 3, available online), a statistically significant association with adenocarcinoma (OR = 1.16; 95% CI = 1.10 to 1.22; *P* = 1.1×10^–8^) was observed among 5164 patients and 38567 control subjects (Figure 3; Supplementary Table 5 and Supplementary Figure 1A, available online). This SNP was not associated with either SCC or small cell carcinoma (*P* ≥ .18) (P_het_ by cell type = 3.9×10^–4^). In a subset of six studies with available data (IARC, MD Anderson Cancer Center (MDACC), MEC, NCI, Samuel Lunenfeld Research Institute study (SLRI), and WHI), when conditioning on the known *TERT* risk variant for lung cancer (rs2736100; linkage disequilibrium [LD] with rs2853676 in European CEU: *r* ^2^ = 0.17), the association with adenocarcinoma was attenuated (OR = 1.06; *P* = .09). Alternatively, the meta-analyzed result among these six studies when not conditioned on rs2736100 was similar to the main adenocarcinoma finding (OR = 1.16; *P* = 1.3×10^–7^).

**Figure 4.**
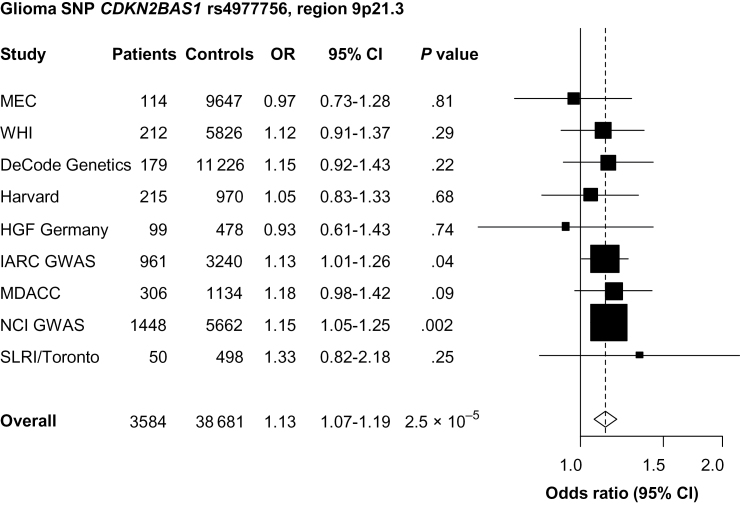
Forest plot of the association between cyclin-dependent kinase 4 inhibitor B antisense RNA 1 (*CDKN2BAS1*) rs4977756 and lung squamous cell carcinoma risk. Study specific and meta-analysis associations are plotted, modeling the G risk allele for glioma. Squares represent odds ratios (ORs); size of the square represents inverse of the variance of the log ORs; horizontal lines represent 95% confidence intervals (CIs); diamonds represent summary estimate combining the study-specific estimates with a fixed-effects model; solid vertical lines represent OR = 1; dashed vertical lines represent the overall ORs. The single-nucleotide polymorphism (SNP) rs4977756 was genotyped in all studies. GWAS = genome-wide association study.

The *CDKN2BAS1* glioma SNP, rs4977756, was not associated with overall lung cancer risk (*P* = .13) but was associated with SCC (OR = 1.11; 95% CI = 1.07 to 1.19; *P* = 2.5×10^–5^) (Figure 4; Supplementary Figure 1B, available online). This SNP was not associated with adenocarcinoma (*P* = .68) or small cell carcinoma (*P* = .48) (*P*_het_ by cell type = .0006) (Supplementary Table 5, available online). Independent effects between rs4977756 and the previously reported lung cancer risk variant in 9p21.3 (11) could not be determined because only a small subset of data on the later variant was available.

Among the 15 nominally statistically significant associations, only two associations were heterogeneous by smoking status (8q24 rs10090154 and 6p21.33 rs6457327) at *P*_het_ < .05 (Supplementary Table 7, available online).

## Discussion

In this large meta-analysis of 18023 lung cancer patients and 60543 control subjects, we examined 165 established cancer risk variants (excluding lung cancer and smoking-related risk variants) and their associations with lung cancer. This is the first study to systematically examine pleiotropic effects from risk variants identified in GWASs of other malignancies on the risk of lung cancer. We found that the breast cancer risk allele “C” of *LSP1* rs3817198 was associated with an increased risk of lung cancer.

*LSP1* encodes the lymphocyte-specific protein 1, an F-actin bundling cytoskeletal protein. In GWAS, common variants in or near the gene have been associated with risk of breast cancer in women (20) and ulcerative colitis in men and women (21). This *LSP1* region is conserved in mice, and studies have found loss of heterozygosity in this region in breast and lung cancers (22,23). We found that this association was stronger in women for overall lung cancer and for adenocarcinoma. When stratifying on both histology and sex, we observed an association in women with adenocarcinoma but not in men with adenocarcinoma. Furthermore, epidemiologic studies of familial aggregation of cancers found an excess of breast cancer among relatives of nonsmokers with lung cancer (24) and relatives of early-onset lung cancer (25), suggesting a genetic susceptibility across these two cancers. To confirm that this association was not a result of excess breast cancer cases, we excluded lung cancer cases with previous history of breast cancer and obtained similar results. The underlying biological mechanism through which *LSP1* may influence cancer development remains to be elucidated. *LSP1* is expressed in lymphocytes, neutrophils, macrophages, and endothelial cells and may regulate neutrophil motility, adhesion to fibrinogen matrix proteins, and transendothelial migration (26).

Risk variants in or near the *TERT-CLPTM1L* locus have been associated with risk of several cancer sites (6), including adenocarcinoma of the lung (6,7,9,27). *TERT* encodes for telomerase reverse transcriptase, which maintains telomere length through each cell division. Telomere shortening is associated with increased genomic instability, thereby increasing the risk of cancer development. The “A” allele of rs2853676, located in intron 2 of *TERT*, was initially reported to be associated with an increased risk of glioma (28). In our study, we found a strongly statistically significant association with adenocarcinoma and notable heterogeneity by histological cell type. Consistent with our findings, the NCI study, which is part of TRICL, reported a modest association between rs2853676 and adenocarcinoma (*P* = 3.4×10^–4^) (7). This same study identified *TERT* rs2736100, also located in intron 2, to be associated with a 12% increase in lung cancer risk (*P* = 1.6×10^−10^) (7). Whereas rs2853676 is in low LD with rs2736100 (European CEU: *r* ^2^ = .17), results from our conditional analysis suggest that the association between rs2853676 and adenocarcinoma may not be independent of rs2736100. In addition, a recent Japanese study found that *TERT* rs2853677 (CEU: *r* ^2^ = 0.59) is associated with lung adenocarcinoma (*P* = 3.1×10^–40^) (29). However, because this SNP was not genotyped in our study, we were unable to condition on rs2853677. It is possible that the association between rs2853676 and adenocarcinoma may be influenced by rs2736100 and rs2853677.

We found that rs4977756 at 9p21.3 was associated with SCC. This SNP is located in *CDKN2BAS1*, a long noncoding RNA region, and near the cluster of two tumor suppressor genes, *CDKN2A* and *CDKN2B*. *CDKN2BAS1* has been implicated in the development of multiple chronic diseases and cancers, due to the role of *CDKN2A* and *CDKN2B* in cell cycle inhibition, senescence, and stress-induced apoptosis (30). Furthermore, three *CDKN2BAS1* spliced variant transcripts expressed in lung cancer cell lines (31) have been shown to have various enhancer activities (32). The SNP rs4977756 has been previously associated with glioma (28,33) and glaucoma (34). A recent meta-analysis of lung cancer GWASs by TRICL found rs1333040, which is approximately 74kb upstream from *CDKN2B*, to be associated with lung cancer (OR = 1.06; *P* = 9.4×10^–5^), with a stronger association for SCC (OR = 1.14; *P* = 2.9×10^–7^) (11). Among European-ancestry populations, there is little LD between rs1333040 and rs4977756 (CEU + Toscans in Italy [TSI]: *r* ^2^ = 0.27). However, because only two studies had genotype data for rs1333040, we were unable to examine the independent effects of the two SNPs. Further evaluation of rs4977756 and SCC risk is needed.

Our finding of pleiotropy between the breast cancer risk locus at *LSP1* and lung cancer risk points toward shared etiologic mechanisms for these two cancer sites. Concurrently, we observed cell type–specific effects for lung cancer with two variants located in cancer pleiotropic regions (*TERT* and risk of lung adenocarcinoma and *CDKN2BAS1* with risk of lung SCC), indicating distinct etiological processes for these two subtypes. These observations of shared and distinct effects with particular genetic loci are consistent with other studies of lung cancer. For example, *EGFR* kinase domain mutations are frequent in lung adenocarcinoma of nonsmokers and extremely rare in lung SCC (35). Alternatively, the *EGFR* variant III mutations have been found in lung SCC and gliomas (36), but not in lung adenocarcinoma (35). These findings demonstrate the complexity of carcinogenesis and the need to study both shared and distinct etiological pathways.

Study limitations include reduced power to detect effects for some of the 165 SNPs. Nonetheless, 72% of the SNPs were genotyped in more than 50% of studies. Due to the limited size of the populations of non-European descent, we were unable to fully examine the generalizability of effects across these populations. Additionally, with the available data, we could only test in a subset of studies the independence of the *TERT* rs2853676 association from the previously reported *TERT* associations. Thus, the associations that we observed with *TERT* rs2853676 and *CDKN2BAS1* rs4977756 may reflect weak LD with previously identified lung cancer risk variants in these regions. However, because the functional SNPs for these regions remain unknown, our findings are informative for future studies (e.g., fine-mapping, functional and population-specific generalizability studies). Furthermore, we recognize the need to study the additional risk loci identified by more recent cancer GWASs. Last, as the majority of our controls excluded all cancers, there may have been a greater likelihood of observing associations with the cancer risk variants studied. However, in the MEC, where control selection allowed inclusion of subjects with other cancers than lung cancer, the associations for the top SNPs were consistent with the overall findings. Our study strengths include the systematic “candidate-SNP” approach based on strong prior evidence of an association from GWASs of cancer, the large sample size from well-characterized epidemiologic lung cancer studies, and the power to examine these associations by cell type, smoking status, and sex.

In conclusion, the breast cancer SNP *LSP1* rs3817198 was associated with lung cancer risk. Our results support the influence of non–lung cancer risk variants on the risk of lung cancer, and these associations may differ by histological cell type and sex. Molecular studies are needed to better characterize these genetic effects and to increase our understanding of the apparent heterogeneity of effects across sex and histological cell type.

## Funding

**PAGE**

This work was supported by the Population Architecture Using Genomics and Epidemiology (PAGE) program that is funded by the National Human Genome Research Institute (NHGRI), supported by U01HG004803 (CALiCo), U01HG004798 (EAGLE), U01HG004802 (MEC), U01HG004790 (WHI), and U01HG004801 (Coordinating Center), and their respective NHGRI ARRA supplements. The contents of this paper are solely the responsibility of the authors and do not necessarily represent the official views of the National Institutes of Health (NIH). The complete list of PAGE members can be found at http://www.pagestudy.org.

The data and materials included in this report result from collaboration between the following studies:

Epidemiologic Architecture for Genes Linked to Environment (EAGLE) is funded through the NHGRI PAGE program (U01HG004798-01 and its NHGRI ARRA supplement). Genotyping services for select NHANES III SNPs presented here were also provided by the Johns Hopkins University under federal contract number (N01-HV-48195) from NHLBI. The study participants derive from the National Health and Nutrition Examination Survey (NHANES), and these studies are supported by the Centers for Disease Control and Prevention (CDC). The findings and conclusions in this report are those of the authors and do not necessarily represent the views of the CDC. The dataset used for the analyses described was obtained from Vanderbilt University Medical Center’s BioVU, which is supported by institutional funding and by the Vanderbilt CTSA grant UL1 TR000445 from NCATS/NIH.

The Multiethnic Cohort study (MEC) characterization of epidemiological architecture is funded through the NHGRI PAGE program (U01HG004802 and its NHGRI ARRA supplement). The MEC study is funded through the National Cancer Institute (R37CA54281, R01 CA63, P01CA33619, U01CA136792, and U01CA98758).

Funding support for the “Epidemiology of putative genetic variants: The Women’s Health Initiative” study is provided through the NHGRI PAGE program (U01HG004790 and its NHGRI ARRA supplement). The WHI program is funded by the National Heart, Lung, and Blood Institute, NIH, US Department of Health and Human Services through contracts HHSN268201100046C, HHSN268201100001C, HHSN268201100002C, HHSN268201100003C, HHSN268201100004C, and HHSN271201100004C. JK is supported by R25CA94880 from NCI. The authors thank the WHI investigators and staff for their dedication, and the study participants for making the program possible. A full listing of WHI investigators can be found at: http://www.whiscience.org/publications/WHI_investigators_shortlist.pdf.

Funding support for the Genetic Epidemiology of Causal Variants Across the Life Course (CALiCo) program was provided through the NHGRI PAGE program (U01HG004803 and its NHGRI ARRA supplement). The following study contributed to this manuscript and is funded by the following agencies: the Atherosclerosis Risk in Communities Study is carried out as a collaborative study supported by National Heart, Lung, and Blood Institute contracts (HHSN268201100005C, HHSN268201100006C, HHSN268201100007C, HHSN268201100008C, HHSN268201100009C, HHSN268201100010C, HHSN268201100011C, and HHSN268201100012C), R01HL087641, R01HL59367 and R01HL086694; NHGRI contract U01HG004402; and NIH contract HHSN268200625226C. The authors thank the staff and participants of the ARIC study for their important contributions. Infrastructure was partly supported by Grant Number UL1RR025005, a component of the NIH and NIH Roadmap for Medical Research. Assistance with phenotype harmonization, SNP selection and annotation, data cleaning, data management, integration and dissemination, and general study coordination was provided by the PAGE Coordinating Center (U01HG004801-01 and its NHGRI ARRA supplement). The National Institute of Mental Health also contributes to the support for the Coordinating Center.

**TRICL**

This work was supported by the Transdisciplinary Research in Cancer of the Lung (TRICL) Study, U19-CA148127 on behalf of the Genetic Associations and Mechanisms in Oncology (GAME-ON) Network.

The SLRI study was supported by Canadian Cancer Society Research Institute (020214), Ontario Institute of Cancer and Cancer Care Ontario Chair Award to RH The ICR study was supported by Cancer Research UK (C1298/A8780 and C1298/A8362—Bobby Moore Fund for Cancer Research UK) and NCRN, HEAL and Sanofi-Aventis. Additional funding was obtained from NIH grants (5R01CA055769, 5R01CA127219, 5R01CA133996, and 5R01CA121197). The Liverpool Lung Project (LLP) was supported by The Roy Castle Lung Cancer Foundation, UK. The ICR and LLP studies made use of genotyping data from the Wellcome Trust Case Control Consortium 2 (WTCCC2); a full list of the investigators who contributed to the generation of the data is available from www.wtccc.org.uk. Sample collection for the Heidelberg lung cancer study was in part supported by a grant (70–2919) from the Deutsche Krebshilfe. The work was additionally supported by a Helmholtz-DAAD fellowship (A/07/97379 to MNT) and by the NIH (U19CA148127). The KORA Surveys were financed by the GSF, which is funded by the German Federal Ministry of Education, Science, Research and Technology and the State of Bavaria. The LUng Cancer in the Young study (LUCY) was funded in part by the National Genome Research Network (NGFN), the DFG (BI 576/2-1; BI 576/2-2), the Helmholtzgemeinschaft (HGF) and the Federal office for Radiation Protection (BfS: STSch4454). Genotyping was performed in the Genome Analysis Center (GAC) of the Helmholtz ZentrumMuenchen. Support for the Central Europe, HUNT2/Tromsø and CARET genome-wide studies was provided by Institut National du Cancer, France. Support for the HUNT2/Tromsø genome-wide study was also provided by the European Community

(Integrated Project DNA repair, LSHG-CT- 2005–512113), the Norwegian Cancer Association and the Functional Genomics Programme of Research Council of Norway. Support for the Central Europe study, Czech Republic, was also provided by the European Regional Development Fund and the State Budget of the Czech Republic (RECAMO, CZ.1.05/2.1.00/03.0101). Support for the CARET genomewide study was also provided by grants from the US National Cancer Institute, NIH (R01 CA111703 and UO1 CA63673), and by funds from the Fred Hutchinson Cancer Research Center. Additional funding for study coordination, genotyping of replication studies and statistical analysis was provided by the US National Cancer Institute (R01 CA092039). The lung cancer GWAS from Estonia was partly supported by a FP7 grant (REGPOT 245536), by the Estonian Government (SF0180142s08), by EU RDF in the frame of Centre of Excellence in Genomics and Estoinian Research Infrastructure’s Roadmap and by University of Tartu (SP1GVARENG). The work reported in this paper was partly undertaken during the tenure of a Postdoctoral Fellowship from the IARC (for MNT). The Environment and Genetics in Lung Cancer Etiology (EAGLE), the Alpha-Tocopherol, Beta-Carotene Cancer Prevention Study (ATBC), and the Prostate, Lung, Colon, Ovary Screening Trial (PLCO) studies and the genotyping of ATBC, the

Cancer Prevention Study II Nutrition Cohort (CPS-II) and part of PLCO were supported by the Intramural Research Program of NIH, NCI, Division of Cancer Epidemiology and Genetics. ATBC was also supported by US Public Health Service contracts (N01-CN-45165, N01-RC-45035 and N01-RC-37004) from the NCI. PLCO was also supported by individual contracts from the NCI to the University of Colorado Denver (NO1-CN-25514), Georgetown University

(NO1-CN-25522), Pacific Health Research Institute (NO1-CN-25515), Henry Ford Health System (NO1-CN-25512), University of Minnesota (NO1-CN-25513), Washington University

(NO1-CN-25516), University of Pittsburgh (NO1-CN-25511), University of Utah (NO1-CN-25524), Marshfield Clinic Research Foundation (NO1-CN-25518), University of Alabama at Birmingham (NO1-CN-75022, Westat, Inc. NO1-CN-25476), University of California, Los Angeles (NO1-CN-25404). The Cancer Prevention Study II Nutrition Cohort was supported by the American Cancer Society. The NIH Genes, Environment and Health Initiative (GEI) partly

funded DNA extraction and statistical analyses (HG-06-033-NCI-01 and RO1HL091172-01), genotyping at the Johns Hopkins University Center for Inherited Disease Research (U01HG004438 and NIH HHSN268200782096C) and study coordination at the GENEVA Coordination Center (U01 HG004446) for EAGLE and part of PLCO studies. Funding for the MD Anderson Cancer Study was provided by NIH grants (P50 CA70907, R01CA121197, R01 CA127219, U19 CA148127, R01 CA55769, K07CA160753) and CPRIT grant (RP100443). Genotyping services were provided by the Center for Inherited Disease Research (CIDR). CIDR is funded through a federal contract from the NIH to The Johns Hopkins University (HHSN268200782096C). The Harvard Lung Cancer Study was supported by the NIH (National Cancer Institute) grants CA092824, CA090578, CA074386.

## Notes

The authors thank the staff and participants of all PAGE and TRICL studies for their important contributions.
